# The Serological Evidence of Positive Rheumatoid Factor in Morphea Patients and Its Relation to Disease Severity

**DOI:** 10.7759/cureus.61105

**Published:** 2024-05-26

**Authors:** Ahmed N Al-Nasrawi, Amenah S Abdulkareem, Mariem N Mohammed Ali

**Affiliations:** 1 Orthopaedic Surgery, Al-Sader Teaching Hospital, Basrah, IRQ; 2 Hematology, College of Medicine - University of Basrah, Basrah, IRQ; 3 Histology, University of Basrah, Basrah, IRQ

**Keywords:** rheumatoid severity, rheumatoid arthritis, autoimmune skin diseases, rheumatoid factor, morphea

## Abstract

Background and objective

Morphea, or localized scleroderma (LS), is an autoimmune skin disorder characterized by inflammation and sclerosis. Its potential causes include infections, genetic predisposition, and trauma. The disease involves cycles of inflammation and fibrosis, leading to skin hardening and scarring, which can cause deformities if untreated. Research exploring the link between morphea and rheumatoid factor (RF), a marker associated with other autoimmune conditions, is ongoing. This study aimed to examine the less-explored role of RF, a marker typically linked to rheumatoid arthritis (RA), in the severity of morphea. It focused on assessing the levels of RF among morphea patients and its correlation with disease severity, intending to provide deeper insights into the condition and its management.

Methods

This study involved a simple randomized cross-sectional analysis to evaluate the role of the RF in measuring morphea severity among patients at the Dermatology and Venereology Department of Al-Sader Teaching Hospital from October 2022 to December 2023. We included participants with clinically and laboratory-confirmed morphea while excluding those with other autoimmune dermatological diseases, recent systemic steroid or immunosuppressive therapy, and pregnant women. The assessment of disease severity was done by utilizing the Localized Scleroderma Cutaneous Assessment Tool (LoSCAT). Statistical analyses were performed using SPSS Statistics version 27 (IBM Corp., Armonk, NY), with a significance threshold of p<0.05.

Results

Elevated RF levels were significantly associated with increased morphea severity, with severe cases showing higher RF levels (mean: 30.34 U/mL) compared to moderate (25.83 U/mL) and mild cases (21.56 U/mL) (p = 0.028). However, no significant correlation was found between RF levels and demographic factors such as age, gender, or occupation. Patients with high RF levels had a longer disease duration (mean: 57.15 years) compared to those with normal levels (25.83 years, p = 0.020). Significant differences were observed in lesion distribution on the back (p = 0.002). Logistic regression indicated that severe morphea patients were more likely to have elevated RF levels [odds ratio (OR): 1.158, p = 0.014].

Conclusions

This study enriches our understanding of RF's role in morphea, revealing no significant correlation with demographic factors but suggesting its potential role in disease chronicity and severity.

## Introduction

Understanding immune system markers in autoimmune diseases such as morphea is crucial for improving their diagnosis and treatment. Morphea, also called chronic localized scleroderma (LS), is characterized by skin and soft tissue inflammation and sclerosis, resulting in skin thickening due to increased collagen in the affected area [1,2]. Epidemiological research indicates that morphea's incidence rate falls between 0.4 and 2.7 cases per 100,000 individuals. It is more common in females and Caucasian individuals, with an equal prevalence among adults and children [3]. The highest rate of incidence in adults is seen in the fifth decade of life, while 90% of cases in children are diagnosed between the ages of 2 and 14 years [4].

Morphea's underlying causes are still unclear and constitute a growing research area. Some studies suggest links to viral or bacterial infections like borreliosis [5,6]. Emerging evidence also suggests a link between morphea and coronavirus disease 2019 (COVID-19), with cases reported following infection. COVID-19 may trigger autoimmune responses, leading to the onset or worsening of morphea [7]. While inconsistent, some associations have also been made between morphea and genetic predisposition [3]. Trauma, including facial injury, and radiation have also been reported as potential activators of morphea [3,8].

The Mayo Clinic Classification categorizes morphea into the following five groups: plaque morphea, generalized morphea (GM), bullous morphea, linear scleroderma, and deep morphea, each having unique clinical manifestations [9]. Plaque morphea, mostly seen in adults, manifests as hard, glossy patches on the skin, often limited to the dermis. It commonly affects the trunk and upper limbs [10]. On the other hand, bullous morphea is a rare variant characterized by the presence of blisters or erosions on the surface of morphea plaques [11]. A solitary lesion on the upper trunk, often near the spine, is characteristic of the deep morphea subtype. It typically appears without preceding inflammation, skin color changes, or hardening [9,12]. Morphea plaques involving more than two body sites are classified as GM [9].

Morphea involves inflammation and fibrosis cycles causing skin thickening, hardening, and scar tissue. If left untreated, it leads to irreversible deformities and functional impairments [13]. Early intervention in morphea involves varied therapies to reduce inflammation, prevent fibrosis, and manage symptoms, while the diagnosis relies on clinical examination and occasionally biopsy to distinguish it from similar conditions [13]. Lab tests, though not definitive for morphea diagnosis, often reveal abnormalities like peripheral blood eosinophilia and changes in aldolase, creatinine kinase, lactate dehydrogenase (LDH), erythrocyte sedimentation rate (ESR), and C-reactive protein (CRP) levels [3]. Several studies have identified various autoantibodies in morphea patients, but their clinical utility for diagnosis or management remains limited [14].

There has been a recent surge of interest in the potential connection between morphea and rheumatoid factor (RF), a marker linked to rheumatoid arthritis (RA) and other autoimmune conditions. RFs, a group of immunoglobulins, have been known for over 70 years and are commonly found in systemic autoimmune disorders like lupus erythematosus, mixed connective tissue disease, polymyositis, and dermatomyositis [15]. This study investigates the association between RF titer and disease severity, clinical implications, and potential therapeutic targets in morphea patients to better understand its role in morphea development.

## Materials and methods

This study was structured as a cross-sectional analysis using a simple random sampling technique. It aimed to assess the RF's role in measuring morphea severity among patients attending the connective tissue clinic of the Department for Dermatology and Venereology, Al-Sader Teaching Hospital, Basrah, Iraq. The study spanned 14 months from October 2022 to December 2023. The methodology adopted sought to elucidate the correlation between RF levels and morphea severity, providing insights into potential diagnostic and prognostic indicators.

All participants with a clinically and biopsy-confirmed diagnosis of morphea by two expert dermatologists were included in this study, while patients with a history of other dermatological autoimmune diseases (such as systemic lupus erythematosus or scleroderma, which could confound the assessment of morphea severity), individuals who had received systemic steroids or immunosuppressive therapy within the last six months (as these treatments could affect the RF levels and disease severity), patients with previous history or recent active RA, and pregnant women (due to the potential for altered immune responses and the risk of complications) were excluded. All participants underwent comprehensive history taking, covering personal details such as age and sex; present history, including onset, course, and duration of lesions; past history with a focus on similar conditions, comorbidities, and other autoimmune diseases; and a family history of related conditions. A thorough general and dermatological examination was conducted for each participant. This included assessing the localization, type of lesion, and the extent of the involved area and skin involvement, thereby providing a detailed characterization of morphea manifestations.

RF levels were measured for all patients using enzyme-linked immunosorbent assay (ELISA), involving a series of precise steps. Initially, microtiter plates were coated with human IgG, to which RF, if present in the patient's serum, would bind. The patient's serum was then added to the plate, and any RF in the sample would attach to the coated IgG. After this, unbound components were washed away, ensuring that only RF bound to the IgG remained. A secondary antibody, typically an anti-human IgM linked to an enzyme such as horseradish peroxidase (HRP), was introduced, which bound specifically to the RF. Subsequently, a substrate for the enzyme, like TMB for HRP, was added. The enzyme catalyzed a reaction that produced a color change, the intensity of which was proportional to the concentration of RF in the sample. This color change was measured using a spectrophotometer, and the results were compared against a standard curve to quantify the RF titer. Additionally, the disease severity was assessed utilizing the Localized Scleroderma Cutaneous Assessment Tool (LoSCAT) (Table 1) [16].

**Table 1:** The Localized Scleroderma Cutaneous Assessment Tool (LoSCAT)* *[16] BSA: body surface area

Category	Features
Mild	BSA, 0%-5%; Involvement of ≤2 anatomical sites; absence of associated functional impairments
Moderate	BSA, 6%-10%; ≥4 morphea plaques that are >3 cm and involve at least 2 of 7 anatomical sites; absence of associated functional impairments
Severe	BSA, >10%; morphea involving the face or crossing joints; the presence of associated functional impairments

Data were collected through a structured, multi-step process at the connective tissue clinic. Initially, patient selection was done using simple random sampling by a computer-generated random sequence after assigning sequential numbers to patients (e.g., 1, 2, 3, 4, 5,.....) and selecting patients randomly. This approach ensured that each patient had an equal and independent chance of being included in the sample, thereby minimizing selection bias and enhancing the representativeness of the sample. At their first visit, the selected patients underwent a comprehensive evaluation that included full history taking and both general and dermatological examinations, ensuring accurate classification according to the study's inclusion and exclusion criteria. Then, blood samples were drawn from all participating patients to measure RF levels, with these tests performed at the hospital’s laboratory to guarantee consistency and reliability. The severity of morphea was then evaluated using LoSCAT, conducted by experienced dermatologists to ensure accurate and unbiased scoring. Finally, all collected data were securely recorded in an electronic database, with each patient assigned a unique identifier to maintain confidentiality and facilitate efficient data analysis, ensuring the integrity of the study findings.

Ethical approval for the study was secured from the Institutional Review Board of Basrah Health Directorate (reference no: 12448). Informed written consent was obtained from all patients after a full explanation of the study's purpose, procedures, and potential benefits. Privacy and confidentiality were rigorously maintained, with each patient assigned a unique privacy code number encompassing all their investigations, ensuring the integrity and ethical conduct of the research. Additionally, to ensure transparency, permission to utilize LoSCAT was obtained from the original publishers.

In this study, a comprehensive statistical approach was used to analyze the research data. Various tests were employed depending on the data size and distribution: Fisher's exact test for small datasets, Pearson Chi-Square test for larger samples, Mann-Whitney U test for non-normal distributions in two independent samples, and Kruskal-Wallis test as a non-parametric alternative to one-way ANOVA. Spearman correlation was used to assess the relationships between ranked or continuous variables, and multinomial logistic regression for predicting categorical outcomes from multiple predictors. The Shapiro-Wilk test was employed to check data normality, guiding the choice of statistical tests to use. All analyses were performed using SPSS Statistics version 27 (IBM Corp., Armonk, NY), with a significance threshold set at a p-value of 0.05 to balance the risks of type I and II errors, ensuring robust and reliable findings.

## Results

Table 2 compares demographic and occupational variables between individuals with normal RF levels and those with high levels. The mean age of individuals with normal levels was 26.04 years, slightly higher than the 22.7 years in those with high levels, but this difference was not statistically significant (p = 0.466). The gender distribution between the two groups was almost identical, with no significant difference noted (p = 0.951). Regarding occupation, students and housewives predominated in both groups, but no significant statistical difference was observed in this regard either (p = 0.478).

**Table 2:** Demographical distribution of the study participants ^*^Fisher's exact test. ^⁑^Pearson Chi-Square test. ^⁂^Mann-Whitney U test RF: rheumatoid factor; SD: standard deviation

Variables		Normal RF (n = 23)	High RF (n = 27)	P-value
Age, years, mean ±SD		26.04 ±17.18	22.7 ±14.96	0.466^⁂^
Sex, n (%)	Male	7 (30.4%)	8 (29.6%)	0.951^⁑^
	Female	16 (69.6%)	19 (70.4%)	
Occupation, n (%)	Employee	3 (13.0%)	5 (18.5%)	0.478^*^
	Student	10 (43.5%)	15 (55.6%)	
	Housewife	10 (43.5%)	7 (25.9%)	

Table 3 highlights the differences in morphea-related characteristics between individuals with normal and high RF levels. The duration of the disease was significantly longer in those with high RF levels, averaging 57.15 years, compared to 25.83 years in those with normal levels (p = 0.020). There was a notable difference in the distribution of lesions on the back, with 39.1% of individuals with normal levels experiencing back lesions versus only 3.7% in the high-level group (p = 0.002). No significant differences were observed in the number, size of lesions, or percentage of body surface area affected in either group. Additionally, functional impairment was not reported in either group. Severity assessments showed that the majority of cases in both groups were categorized as moderate, with no significant differences observed except in the severe category.

**Table 3:** Morphea-related data distribution based on RF levels ^*^Independent t-test.^ ⁑^Pearson Chi-Square test RF: rheumatoid factor; SD: standard deviation

Variables		Normal RF (n = 23)	High RF (n = 27)	P-value
Duration of the disease, years, mean ±SD		25.83 ±25.08	57.15 ±57.79	0.020^*^
Anatomical site, n (%)	Head and neck	4 (17.4%)	10 (37.0%)	0.123^⁑^
	Upper limp	5 (21.7%)	7 (25.9%)	0.730^⁑^
	Chest	7 (30.4%)	10 (37.0%)	0.623^⁑^
	Back	9 (39.1%)	1 (3.7%)	0.002^⁑^
	Abdomen	3 (13.0%)	5 (18.5%)	0.599^⁑^
	Lower limb	7 (30.4%)	7 (25.9%)	0.723^⁑^
Number of skin lesions, n (%)	Singe lesion	11 (47.8%)	13 (48.1%)	0.982^⁑^
	Multiple lesions	12 (52.2%)	14 (51.9%)	
Size of the skin lesion, cm, mean ±SD		3.30 ±1.14	2.85 ±1.29	0.20^*^
Percentage of the body surface area covered, mean ±SD		3.04 ±1.49	2.59 ±1.33	0.265^*^
Functional impairment, n (%)	Yes	0 (0.0%)	0 (0.0%)	------
	No	23 (100.0%)	27 (100.0%)	
Severity of the disease, n (%)	Mild	6 (26.1%)	6 (22.23%)	0.534^⁑^
	Moderate	13 (56.5%)	13 (48.15%)	
	Severe	4 (17.4%)	8 (29.62%)	

**Figure 1:**
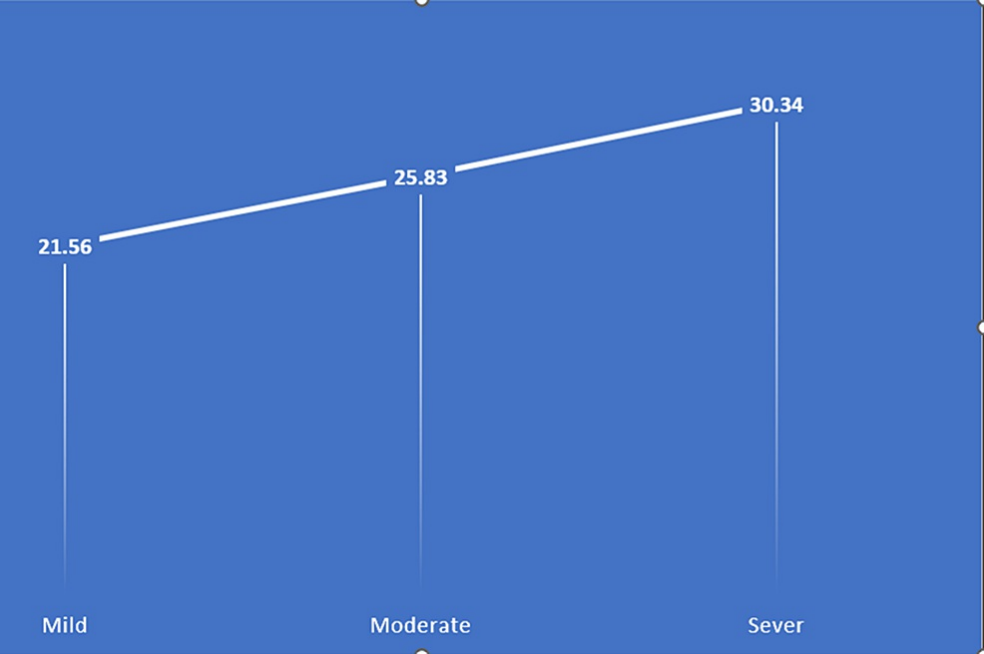
Image depicting morphea severity by RF levels X-axis: morphea severity as assessed by LoSCAT. Y-axis: RF levels LoSCAT: Localized Scleroderma Cutaneous Assessment Tool; RF: RF: rheumatoid factor

As shown in Table 4, there was a statistically significant correlation between RF levels and morphea severity, (p = 0.028). The data showed that individuals with severe morphea had the highest mean RF level at 30.34 U/mL (±11.63), followed by those with moderate morphea at 25.83 U/mL (±7.53) and those with mild morphea at 21.56 U/mL (±5.12). This trend suggests that higher RF levels may be associated with increased severity of morphea, underscoring the importance of further studies to explore this relationship more deeply (Figure 1).

**Table 4:** Severity of morphea distribution based on RF levels ^*^Kruskal-Wallis test RF: rheumatoid factor; SD: standard deviation

Variables	Mild (n = 16)	Moderate (n = 24)	Severe (n = 10)	P-value
RF level, U/mL, mean ±SD	21.56 ±5.12	25.83 ±7.53	30.34 ±11.63	0.028^*^

Table 5 illustrates the relationship between the size of skin lesions, the percentage of body surface area covered, and RF levels using Spearman correlation coefficients. The analysis showed a negative correlation between the size of the skin lesions and RF levels with a coefficient of -0.207, suggesting that larger lesions correlate with lower RF levels; however, this relationship was not statistically significant (p = 0.148). Similarly, the percentage of the body surface covered area had a Spearman correlation coefficient of -0.100 with RF levels, also indicating a negative correlation. Similar to the lesion size, this relationship was not statistically significant either (p = 0.491).

**Table 5:** The correlation of the size of the lesion and the percentage of the body surface area covered with RF levels RF: rheumatoid factor; SD: standard deviation

Variables	Spearman correlation	P-value
Size of the skin lesion, cm, mean ±SD	-0.207	0.148
Percentage of the body surface area covered, mean ±SD	-0.100	0.491

Table 6 presents the results of the logistic regression analysis exploring the relationship between morphea severity and RF levels across three severity categories: mild, moderate, and severe. The analysis highlights that individuals with severe morphea were significantly more likely to have elevated RF levels, with an odds ratio of 1.158 and a p-value of 0.014, suggesting a strong association. Conversely, individuals with mild morphea showed a decreased likelihood of high RF levels, indicated by an odds ratio of 0.864 and a p-value of 0.014. However, for those with moderate morphea, the association was not statistically significant, as reflected by an odds ratio of 1.087 and a p-value of 0.093.

**Table 6:** Logistic regression test of the severity of morphea with RF levels CI: confidence interval; RF: rheumatoid factor

Morphea severity	Coefficient regression	Odds ratio	P-value	95% CI
Mild	-0.146	0.864	0.014	0.76-0.97
Moderate	0.083	1.087	0.093	0.98-1.19
Severe	0.146	1.158	0.014	1.03-1.3

## Discussion

Our findings highlight a complex relationship between RF levels and morphea characteristics. Notably, the prolonged duration of disease in patients with high RF levels suggests a potential role for RF in the chronicity of morphea. This is an intriguing association, considering that RF is traditionally linked to RA and other autoimmune conditions [17]. The term "rheumatoid factor", despite its association with RA, extends beyond RA-specific markers [18]. RFs are commonly produced as part of immunization responses and secondary immune reactions to infections, playing a role in pathogen elimination. Conditions like systemic lupus erythematosus, Sjogren's disease, and sarcoidosis also exhibit an association with RF [19].

Moreover, the statistically significant correlation between elevated RF levels and increased severity of morphea adds a new dimension to our understanding of this skin condition. The data suggests that higher RF levels may indicate a more severe disease, with individuals having severe morphea also showing the highest mean RF levels. In a 2004 study by Mimura et al. [20], involving 43 Japanese patients diagnosed with LS, notable findings emerged. The study observed markedly elevated serum levels of all three isotypes in patients with GM, in comparison to healthy subjects. GM is the most severe manifestation within the spectrum of LS. It manifests as extensive skin engagement characterized by numerous hardened plaques, hyperpigmentation, and frequent muscle atrophy [21,22]. This outcome aligns with our understanding that GM is more likely to exhibit immunological abnormalities, as established in prior literature [21]. Furthermore, in a separate investigation by Takehara et al. [23], the researchers studied RF in the context of LS and evaluated its potential cross-reactivity with antihistone antibodies. By employing a latex agglutination test, the presence of IgM rheumatoid factor was identified in 60% of 20 patients diagnosed with LS and in 82% of those afflicted with GM. Furthermore, an absorption test aimed at assessing the activity of RF with human IgG showed no evidence of cross-reactivity between antihistone antibodies and RF [23].

It is also important to mention that several studies have consistently found a higher frequency of association between RF and arthritis or other extracutaneous manifestations [24]. The incidence of co-existing inflammatory arthritis, in particular, may be as high as 20.8%, as stated in a recent cohort of 53 patients diagnosed with LS, by Kashem et al. [25]. In morphea, the presence of extracutaneous manifestations is linked to higher damage and impact scores, as well as a greater extent of cutaneous disease. Notably, these patients tend to have a prolonged persistence of active disease despite receiving more systemic immunosuppressive treatment, which is associated with functional impairment, affecting approximately 27-38% of patients [26,27]. Moreover, the presence of extracutaneous involvement increases the requirement for immunosuppressants such as methotrexate and glucocorticoids. It is also associated with elevated pain scores and a lower quality of life [27,28]. Considering the findings mentioned earlier, the observed associations between extracutaneous involvement, disease impact, and treatment outcomes underscore the potential role of RF as an indicator of not only the extent of cutaneous involvement but also the overall severity and persistence of the disease.

Our analysis of the relationship between RF levels and the clinical features of morphea revealed weak negative correlations. Specifically, the observed Spearman correlation coefficients of -0.207 and -0.100, concerning the size of skin lesions and the percentage of body surface area affected, respectively, suggest a potential inverse relationship. That is, as RF levels increase, the size of skin lesions and the affected body surface area may tend to decrease. However, these correlations did not reach statistical significance (p = 0.148 and 0.491, respectively). A study by Mimura et al. [20] demonstrated a significant increase in the number of sclerotic lesions among patients with IgM RF compared to those without, pointing to a possible linear relationship between the number of skin lesions and RF levels. They also explored the potential use of IgM RF as a marker for gauging disease severity [20]. Moreover, Sato et al. [21] have stated that in individuals with linear LS affecting the extremities, elevated RF levels may be detected and occasionally show a correlation with the clinical severity of disease activity, especially if they have concurrent joint involvement [21]. Similarly, several studies have stated that aldolase, creatinine phosphokinase, LDH, and CRP, in addition to RF, are helpful in the assessment of the activity of LS [29,30].

The findings in the present study appear to contradict established literature regarding the relationship between RF levels and the size of skin lesions as well as the body surface area affected in cases of morphea. However, it is worth mentioning that the absence of statistical significance may suggest that while there is a tendency for these variables to move in opposite directions, the relationship is not strong enough to confirm that changes in one are reliably associated with changes in the other within this population.

The study's limitations primarily pertain to its small and demographically narrow sample size, which could limit the generalizability of the findings. The cross-sectional design, focusing on a single point in time, might have failed to capture the disease's progression or the fluctuating nature of RF levels. Additionally, excluding patients with other autoimmune diseases could have led us to overlook the complexity of autoimmune interactions. Lastly, the study's geographical limitation (it was conducted at a single clinic in Basrah, Iraq) means that its results may not be representative of the wider, diverse population suffering from morphea, potentially affecting the external validity of the results.

## Conclusions

The findings of this study enrich our understanding of the role of RF in morphea, revealing no significant correlation with demographic factors but suggesting its potential involvement in disease severity. These findings highlight the need for a reevaluation of RF beyond traditional associations, involving more nuanced investigations into its implications for morphea management and treatment strategies by analyzing the condition's severity among the patients.
